# 129 Prioritising health in mobility planning: unlocking the health protential of Sustainable Urban Mobility Plans (SUMPs)

**DOI:** 10.1093/eurpub/ckae114.103

**Published:** 2024-09-26

**Authors:** Sonja Kahlmeier, Dena Kasraina, Hannah E Murdock, Ahmadreza Faghih Imani, Yurong Yu, Audrey de Nazelle, Dominic Stead

**Affiliations:** Swiss Distance University of Applied Science, Department of Health, Zurich, Switzerland; Eindhoven University of Technology, Department of the Built Environment, Eindhoven, The Netherlands; Imperial College London, Centre for Environmental Policy, London, United Kingdom; Imperial College London, Centre for Environmental Policy, London, United Kingdom; Imperial College London, Centre for Environmental Policy, London, United Kingdom; Imperial College London, Centre for Environmental Policy, London, United Kingdom; Aalto University, School of Engineering, Espoo, Finland

## Abstract

**Purpose:**

Urban mobility can have detrimental impacts on health and quality of life, but can also be an opportunity for health promotion, e.g. through walking and cycling. While health impacts of transport are well known, the extent to which health is considered in mobility plans is less obvious. European cities are strongly encouraged to develop Sustainable Urban Mobility Plans (SUMPs).

**Methods:**

We assess the extent to which health is reflected in SUMPs in cities, with regards to: i) key health and health equity terminology, ii) how explicit transport pathways to health are made, and iii) whether health is operationalised into targets and key performance indicators (KPIs) and iv) how well elaborated the health-rationale of various actions and measures is . We analysed the latest SUMPs in the Eltis City database of urban mobility plans with a quantitative text analysis, supported by the development of a health dictionary and a policy analysis checklist. We carried out a qualitative analysis of a purposive sub-sample to verify the validity of the quantitative approach and provide further nuance to the assessment.

**Results:**

230 SUMPS (2006-2023) from 31 countries were usable for quantitative analysis, from which 13 were included into qualitative analysis, reflecting a range of city sizes, countries, and focus on health. The findings show that while health is often touched upon, and its prominence seems to be increasing, SUMPs miss out on the opportunity to embrace mobility as a driver of health promotion. The link between transport and equity and social and mental wellbeing is not frequently discussed. Detailed targets and KPIs for several health pathways are scarce or missing, as are the health rationale and outcomes for proposed measures. Health aspirations are concerned with minimising detrimental impacts of transport on health, primarily from traffic injuries and to a lesser extent from air pollution. Concepts such as accessibility and active travel feature prominently but are not explicitly identified as an opportunity to enhance health.

**Conclusions:**

Urban mobility planning across Europe miss an opportunity to embrace health as a means to engage across sectors and society to help promote transformative urban sustainability policies.

